# *Prss55* but not *Prss51* is required for male fertility in mice^†^

**DOI:** 10.1093/biolre/ioaa041

**Published:** 2020-04-17

**Authors:** Kiyonori Kobayashi, Tsutomu Endo, Takafumi Matsumura, Yonggang Lu, Zhifeng Yu, Martin M Matzuk, Masahito Ikawa

**Affiliations:** 1 Research Institute for Microbial Diseases, Osaka University, Suita, Osaka, Japan; 2 Graduate School of Frontier Biosciences, Osaka University, Suita, Osaka, Japan; 3 Immunology Frontier Research Center, Osaka University, Suita, Osaka, Japan; 4 Graduate School of Pharmaceutical Sciences, Osaka University, Suita, Osaka, Japan; 5 Center for Drug Discovery and Department of Pathology & Immunology, Baylor College of Medicine, Houston, Texas, USA; 6 Graduate School of Medicine, Osaka University, Suita, Osaka, Japan; 7 The Institute of Medical Science, The University of Tokyo, Minato-ku, Tokyo, Japan

**Keywords:** male infertility, sperm, testes, epididymides, uterotubal junction, ADAM3

## Abstract

Mammalian spermatozoa are produced in the testis through spermatogenesis and matured in the epididymis to acquire fertilizing ability. Spermatozoa are ejaculated and migrate from the uterus to the oviducts to fuse with oocytes. Although over 2000 genes are expressed abundantly in mouse testes, the genes responsible for male fertility are not yet fully clarified. Here, we focused on two testis-enriched serine protease genes, *Serine protease (Prss) 51* and *Prss55*, which overlap their gene loci partially in both mice and humans. To characterize their functions in male fertility, we first generated *Prss51* and *Prss55* double knockout (DKO) mice by CRISPR/Cas9 system and found that the DKO mice were sterile. DKO spermatozoa exhibit impaired migration from the uterus to the oviduct and impaired ability to bind the zona pellucida (ZP) of oocytes. Moreover, a sperm membrane protein, ADAM3 (a disintegrin and metalloprotease 3), which plays a role in sperm migration through uterotubal junction (UTJ) and sperm–ZP binding, disappeared in the DKO spermatozoa from the epididymis. We next generated single knockout (KO) mice lacking *Prss51* and found that *Prss51* KO mice are fertile. We also generated single KO mice lacking *Prss55* and found that *Prss55* KO mice phenocopy the DKO mice, demonstrating impaired sperm migration and sperm–ZP binding and a severe defect in fertility. We conclude that *Prss55*, but not *Prss51*, is required for male fertility in mice, by stabilizing ADAM3 protein for efficient sperm–UTJ migration and sperm–ZP binding. Our findings have implications for understanding additional genetic causes of the idiopathic male infertility and for the development of male or female contraceptives.

## Teaser

The testis-specific serine protease gene *Prss55*, but not *Prss51*, is required for sperm ADAM3 maturation and male fertility in mice; *Prss55* KO sperm fail both to migrate from the uterus into the oviduct and to bind the ZP of oocytes.

## Introduction

Mammalian spermatozoa are produced in the seminiferous tubules of the testis through the process of spermatogenesis. During spermatogenesis, diploid spermatogonia give rise to haploid spermatozoa via meiosis [1]. These spermatozoa are released into the lumen of the seminiferous tubules and undergo a maturation process to acquire their motility and fertility as they migrate through the head (caput), body (corpus), and distal (cauda) epididymis [2]. Finally, spermatozoa are ejaculated, undergo capacitation in the female reproductive tract, and migrate into the oviducts to fuse with oocytes [3, 4] to form embryos. Although over 2000 genes are expressed abundantly in the mouse testis [5–7], the genes responsible for sperm function and fertilization are not yet fully clarified. Thus, functional analysis of the testis-enriched genes is necessary to understand the male fertility and develop male or female contraceptives.

In the present study, we focused on two testis-enriched serine protease genes, *Serine protease (Prss) 51* and *Prss55*, which are present in mice and humans [8, 9]. Serine proteases constitute nearly one-third of all proteases [10, 11], and several serine proteases are expressed predominantly in the testis and play important roles in sperm function and fertilization [8, 12–15]. A putative trypsin-like serine protease, PRSS37, is exclusively expressed in testicular germ cells during late spermatogenesis and required for fertility in mice [15]. Mice lacking another serine protease gene, *Prss21*, are subfertile; their spermatozoa exhibit decreased motility, morphological malformation, and impaired ability to bind zona pellucida (ZP) of oocytes in vitro [13, 14].

*Prss51* and *Prss55* genes, which encode putative trypsin-like serine proteases [8, 9], are located on the same chromosome adjacent to each other and overlap their gene loci partially, both in mice and humans. PRSS55 is a predicted Glycosylphosphatidylinositol (GPI)-anchored membrane protein, specifically expressed in the mouse testis [8]. A recent study has shown that mice lacking *Prss55* exhibit severe male infertility; *Prss55* KO spermatozoa fail both to migrate from the uterus into oviduct in vivo and to bind ZP of oocytes in vitro [8]. However, another research group has shown that *Prss55* KO male mice are fertile in vivo and thus concluded that *Prss55* is dispensable for male fertility [9]. So far, it remains unclear whether *Prss55* is required for male fertility. It should be noted that *Prss51*, the gene overlapping with *Prss55* locus, may play some roles in male fertility and explain the discrepancy.

In the present study, we generated gene knockout (KO) mice by the CRISPR/Cas9 system to characterize the functions of *Prss51* and *Prss55* on male fertility. Specifically, we first generated double KO (DKO) mice lacking both *Prss51* and *Prss55* and analyzed male fertility. We then generated single KO mice lacking either *Prss51* or *Prss55* to clarify the requirement of each gene for male fertility.

## Materials and methods

### Animals

All animal experiments were approved by the Animal Care and Use Committee of the Research Institute for Microbial Diseases, Osaka University. Mice were maintained under a 12-h light/dark cycle (lights on from 8:00 to 20:00). All wild-type C57BL6, B6D2F1/J, and Institute of Cancer Research (ICR) mice were purchased from Japan SLC (Shizuoka, Japan). In this study, we generated genetically modified mouse lines, *Prss51* and *Prss55* DKO mice, *Prss51* single KO, and *Prss55* single KO mice. *Prss51* single KO mice (STOCK-*Prss51* <em1Osb>; RBRC11051 and CARD2958) and *Prss55* single KO mice (STOCK-*Prss55* <em1Osb>; RBRC11052 and CARD2959) were deposited to the RIKEN BioResource Research Center (http://mus.brc.riken.jp/en/) and the Center for Animal Resources and Development (CARD), Kumamoto University (http://card.medic.kumamoto-u.ac.jp/card/english/). *Prss51* and *Prss55* DKO mice will be deposited. *Prss51* and *Prss55* heterozygous (Het) DKO mice were crossed with RBGS (Red Body Green Sperm) transgenic mice [16], in which spermatozoa exhibit enhanced green fluorescent protein (EGFP) in the acrosome and red fluorescence (DsRed2) in the mitochondria within the flagellar midpiece, to generate the DKO mice carrying RBGS transgene after subsequent breeding.

### RT-PCR analysis

Mouse complementary DNA (cDNA) was prepared from multiple adult tissues of wild-type C57BL6 mice [17]. Briefly, using TRIzol reagent (Invitrogen, USA), total RNA was isolated from multiple adult tissues of wild-type mice and multiple adult human tissues obtained from the Human Tissue Acquisition & Pathology core service (Baylor College of Medicine, USA). Mouse and human cDNA were prepared using SuperScript III Reverse Transcriptase (Invitrogen, USA) following the manufacturer’s instruction. The amplification conditions were 2 min at 94°C, followed by 30–35 cycles of 94°C for 30 s, 65°C for 30 s, and 72°C for 30 s, with a final 5–7 min extension at 72°C. The primers used are listed in Supplementary Table S1.

### Generation of KO mice with the CRISPR/Cas9 system

*Prss51* and *Prss55* DKO mice were generated by transfection of pX459 plasmid (https://www.addgene.org/62988/) into mouse embryonic stem (ES) cells using a procedure described previously [18, 19]. The mutant ES cells were aggregated with 8- or 16-cell stage embryos, and then the developed blastocysts were transferred into the uterus of embryonic day 2.5 pseudopregnant ICR females. Contribution of ES cells to germ cell lineage was confirmed by EGFP signal because the ES cells ubiquitously express EGFP in the cytoplasm of all cell types and the acrosome (anterior half of the head) of spermatozoa [20]. *Prss51* and *Prss55* single KO mice were generated by introducing guide RNAs (gRNAs) and the CAS9 enzyme (Thermo Fisher Scientific) into fertilized eggs with an electroporator (NEPA21, Nepagene)*.* A search for guide RNA (gRNA) on-target and off-target sequences was performed using CRISPRdirect software (https://crispr.dbcls.jp/) [21]. The gRNA target sequences used for *Prss51* and *Prss55* DKO mice were: 5′-GAGGAACGCTGACTACCGGT-3′ for the last exon of *Prss51* single guide RNA (sgRNA#1) and 5′-GCACACTGTAACTTAGGGTT-3′ for the last exon of *Prss55* (sgRNA#2) (Figure 2A). The gRNA sequences used for *Prss51* single KO mice were: 5′-TGAGCAGTGCAATTAGAAGT-3′ for the second exon (sgRNA#3) and 5′-GAGGAACGCTGACTACCGGT-3′ for the last exon (sgRNA#1) (Figure 4A). The gRNA sequences used for *Prss55* single KO mice were: 5′-CTAGCTCAGGACACGTCCTT-3′ for the fifth exon (sgRNA#4) and 5′-GCACACTGTAACTTAGGGTT-3′ for the last exon (sgRNA#2) (Figure 5A). Screening of the obtained mutant mice was performed by polymerase chain reaction (PCR) and subsequent sequencing of the PCR product.

### PCR analysis (genotyping)

The primers used are listed in Supplementary Table S2. Detailed genotype information of mutant mouse lines is shown in Figures 2C, 4C, and 5C.

### Phylogenetic trees and protein multiple sequence alignment

Phylogenetic trees and protein multiple sequence alignment were constructed by using GENETYX.

### Male fertility test

Sexually mature mutant male mice were caged with 2-month-old B6D2F1 females (SLC, Japan) for several months, and the number of pups in each cage was counted. Fertility is presented as the number of total pups divided by the number of vaginal plugs in females.

### Testis histology and sperm morphology

After breeding studies, males were sacrificed by cervical dislocation following anesthesia. Testes were weighed individually, then fixed overnight in Bouin solution, and were processed for paraffin embedding. Paraffin sections were cut at 5 μm, stained with periodic acid-Schiff, and then counterstained with Mayer hematoxylin solution (Wako). The cauda epididymal spermatozoa were dispersed in phosphate-buffered saline (PBS), and then sperm morphology was observed under a phase-contrast microscope (BX50, Olympus).

### Sperm motility analysis

Cauda epididymal spermatozoa were dispersed in TYH (Toyoda, Yokoyama, Hoshi) drops for sperm motility. After an incubation period of 10 and 120 min, the sperm motility pattern was examined using the CEROS II sperm analysis system (software version 12.3; HamiltonThorne Biosciences) [22] and the default program (Mouse CytoD ×4 dark-field settings) of the CEROS II sperm analysis system (software version 1.5.2; Hamilton Thorne Biosciences).

### In vitro fertilization

Mouse in vitro fertilization (IVF) was performed as described previously [23]. Briefly, oocytes with cumulus cells (cumulus–oocyte complexes [COCs]) were collected from superovulated females 14 h after Human chorionic gonadotropin (hCG) injection and placed in 100 μL drops of TYH medium covered with paraffin oil. Epididymal spermatozoa were collected from male mice and incubated in TYH medium for 2 h for capacitation. Capacitated spermatozoa were added to each drop containing COCs at a final concentration of 2 × 10^5^ spermatozoa/mL. To remove cumulus cells, COCs were treated with hyaluronidase (1 mg/mL) for 5 min. To remove ZP, COCs were treated with collagenase (1 mg/mL) for 5 min. After 8 h of incubation, the formation of pronuclei was observed under a Hoffman modulation contrast microscope.

### Sperm–ZP-binding assay

The sperm–ZP-binding assay was performed as described previously [24]. Briefly, 30 min after mixing with 2-h–incubated spermatozoa, cumulus-free oocytes were fixed with 0.2% glutaraldehyde. The bound spermatozoa were observed with an Olympus IX73 microscope.

### Immunoblot analysis

Immunoblot analysis was performed as described previously [25]. Briefly, testes and epididymides were collected from mice. Spermatozoa were collected from caput, corpus, and cauda epididymis, respectively. These samples were homogenized in lysis buffer containing 1% Triton X-100 and 1% protease inhibitor (Nacalai Tesque, Kyoto, Japan) and then were centrifuged (10 000 *g* for 20 min at 4°C, and the supernatants were collected. Protein lysates were separated by SDS/PAGE under reducing condition and transferred to polyvinylidene difluoride (PVDF) membranes (Merck Millipore). After blocking, blots were incubated with primary antibodies overnight at 4°C and then incubated with secondary antibodies conjugated with horseradish peroxidase. The detection was performed by using Chemi-Lumi One Super (Nacalai Tesque). Antibodies and incubation conditions are provided in Supplementary Table S3.

### Imaging of sperm migration through the female reproductive tract

Live imaging of spermatozoa inside the female reproductive tract was conducted as previously described [16]. B6D2F1 female mice > 8 week old were superovulated and mated with male mice carrying the RBGS transgene 12–14 h after hCG injection. We checked for vaginal plugs every 30 min and sacrificed the female mice 2–3 h after observing a vaginal plug. The female reproductive tracts were subsequently dissected, and the spermatozoa inside the tract were observed using a BZ-X710 microscope.

### Statistical analysis

Statistical analyses were performed using Student *t* test inserted into Microsoft Excel after the data were tested for normality of distribution. Differences were considered significant at *P* < 0.05 and *P* < 0.01.

## Results

### Expression patterns of *Prss51* and *Prss55*

**Figure 1:**
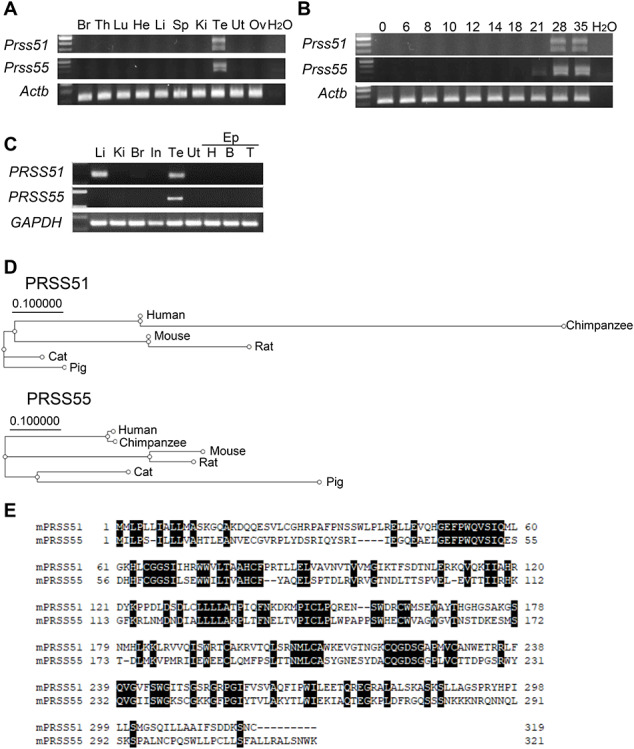
Characterization of *Prss51* and *Prss55.* A. Expression of mouse Prss51 and Prss55 was examined by RT-PCR using RNA isolated from various organs. *Actin beta* (*Actb*) was used as a control. Br, brain; Th, thymus; Lu, lung; He, heart; Li, liver; Sp, spleen; Ki, kidney; Te, testis; Ut, uterus; Ov, ovary. B. Expression of mouse Prss51 and Prss55 was examined by RT-PCR using RNA isolated from the testis at the indicated postnatal days. *Actb* was used as a control. C. Expression of human PRSS51 and PRSS55 was examined by RT-PCR using RNA isolated from various organs. *GAPDH* (Glyceraldehyde-3-Phosphate Dehydrogenase), was used as a control. Li, liver; Ki, kidney; Br, brain; In, intestine; Te, testis; Ut, uterus; Ep, epididymis; EP (H), head; EP (B), body; EP (T), tail. D. Phylogenetic trees of PRSS51 and PRSS55. E. Alignment of the predicted amino acid sequence of mouse PRSS51 and PRSS55. Black boxes indicate an identical match between these proteins.

We examined the expression patterns of *Prss51* and *Prss55* by reverse transcription PCR (RT-PCR) with multiple tissues in mice and humans, or with the testes from different ages in mice. We found that *Prss51* and *Prss55* were specifically expressed in mouse testes (Figure 1A) from postnatal day 28 (Figure 1B) when elongating or elongated spermatids first appear in the testis [26]. In humans, *PRSS51* was predominantly expressed in testes and liver, and *PRSS55* was specifically expressed in testes (Figure 1C). The protein sequence of PRSS51, as well as PRSS55 [9], is highly conserved among various species in mammals, including mice and humans (Figure 1D and Supplementary Figure S1A and B). Alignment of the predicted amino acid sequence of mouse PRSS51 and PRSS55 exhibited 34.2% sequence identity and 54.2% sequence similarity (Figure 1E).

### Generation of *Prss55* and *Prss51* double KO mice

**Figure 2:**
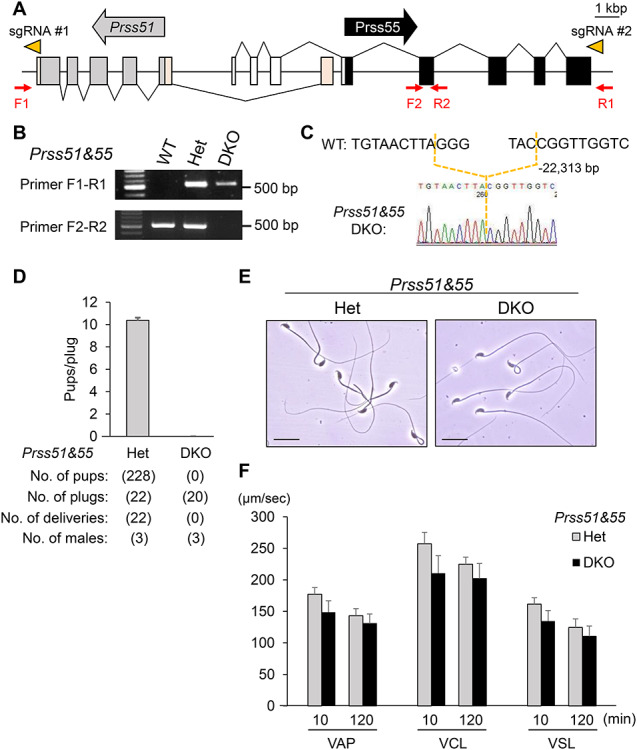
Male lacking both *Prss51* and *Prss55* are sterile. A. Genomic structure and deletion strategy for creation of mouse *Prss51* and *Prss55* DKO mice. The direction of the arrows, in gray (*Prss51*) and black (*Prss55*), indicates the direction of transcription. The direction of the arrowheads (orange) indicates the direction of sgRNA (#1 and #2). Gray boxes, *Prss51* coding region; light orange boxes, *Prss51* noncoding region; black boxes, *Prss55* coding region; white boxes, *Prss55* noncoding region. F1 and F2, forward primers for genotyping; R1 and R2, reverse primers for genotyping. Scale bar, 1 kbp. B. Genotyping with genomic PCR. Primers shown in panel A were used for the PCR. WT, wild type; Het, heterozygous; DKO, *Prss51* and *Prss55* homozygous DKO. C. DNA sequencing. The sequence of PCR amplicon was analyzed. A region of 22,313 bp was deleted in *Prss51* and *Prss55* DKO mice. D. Fertility is presented as pups/plug: the number of total pups born divided by the number of vaginal plugs in females after natural mating. (10.4 ± 0.25 for wild-type females mated with the Het males, and 0 with the DKO males). Error bars, mean ± SD. E. Morphology of cauda epididymal spermatozoa in *Prss51* and *Prss55* DKO mice. Scale bars, 20 μm. F. Sperm motility analysis after an incubation period of 10 and 120 min. There was no significant difference in sperm motility parameters between Het and DKO spermatozoa (*P* > 0.05). VAP, average path velocity; VSL, straight line velocity; VCL, curvilinear velocity.

Both *Prss51* and *Prss55* are located on mouse chromosome 14qD1 locus, with partially overlapping their gene loci (Figure 2A). *Prss51* is composed of six exons and encodes a 319-amino acid protein, and *Prss55* is composed of eight exons and encodes a 321-amino acid protein (Figures 1E and 2A). To study the functions of *Prss51* and *Prss55*, we deleted both genes in ES cells by transfecting two plasmids expressing sgRNA#1, sgRNA#2, and CAS9 (Figure 2A). We generated chimeric mice from an ES cell clone carrying a homozygous double deletion of *Prss51* and *Prss55* (Supplementary Figure S2A and B). We confirmed the contribution of the ES cells to germ cell lineage in the chimeric mice (Supplementary Figure S2C) and obtained *Prss51* and *Prss55* DKO mice from Het F1 intercrosses. The genotype of the DKO mice was confirmed by genomic PCR (Figure 2B) and direct sequencing (Figure 2C).

### Phenotypes of male mice lacking both *Prss51* and *Prss55*

We evaluated the fertility of *Prss51* and *Prss55* double Het and double homozygous KO (DKO) male mice (Figure 2D). The pregnancy rate (the number of deliveries per the number of the vaginal plugs) was 100% (22/22) for wild-type female mice mated with the Het male mice, and 0% (0/20) for wild-type females mated with the DKO males (Figure 2D). The average number of pups divided by the number of vaginal plugs was 10.4 ± 0.25 for the double Het males (*n* = 3), and 0 for the DKO males (*n* = 3) (Figure 2D). We found that there was no difference in testicular morphology, weight, and histology (Supplementary Figure S3A–C), and in sperm morphology and motility (Figure 2E and F), between the Het and DKO males. Thus, *Prss51* and *Prss55* DKO males are sterile.

**Figure 3:**
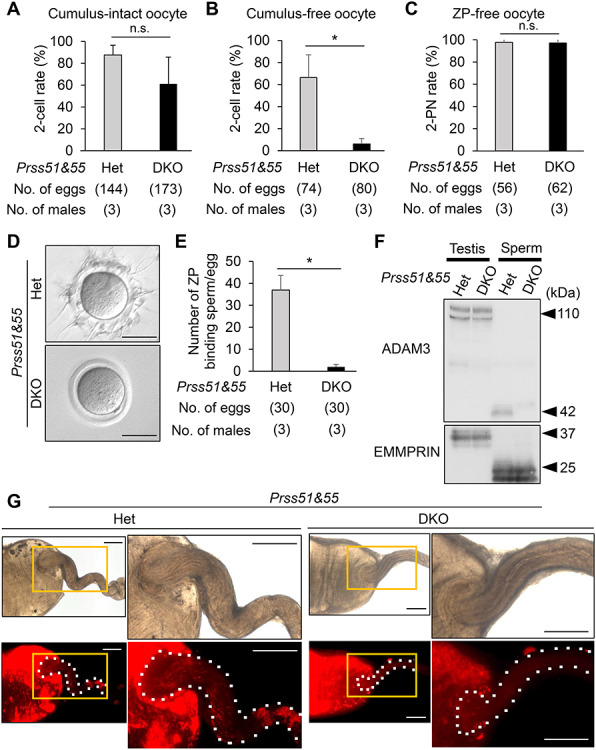
Phenotypic analysis of mouse spermatozoa lacking both *Prss51* and *Prss55*. A. IVF rates (percentage of two-cell stage eggs) in cumulus-intact oocyte using spermatozoa derived from *Prss51* and *Prss55* heterozygous (Het) and homozygous (DKO) males. Average IVF rates of Het and DKO spermatozoa were 87.6 ± 8.9 and 61.1 ± 24.5%, respectively. N.s., no significance (*P* > 0.05). Error bars, mean ± SD. B. IVF rates (percentage of two-cell stage eggs) in cumulus-free oocyte using spermatozoa from Het and DKO mice. Average IVF rates of Het and DKO spermatozoa were 66.3 ± 20.6 and 6.0 ± 5.0%, respectively. *P < 0.05. Error bars, mean ± SD. C. IVF rates (percentage of two pronuclei [2PN] eggs) in ZP-free oocytes using spermatozoa from Het and DKO mice. Average IVF rates of spermatozoa from Het and DKO mice were 97.8 ± 3.2 and 97.1 ± 4.0%, respectively. N.s., no significance (*P* > 0.05). Error bars, mean ± SD. D. Sperm–ZP-binding assay. Spermatozoa from Het and DKO mice were used to inseminate cumulus-free oocytes. Scale bars, 50 μm. E. Average numbers of ZP-binding spermatozoa per total number of eggs in Het and DKO male mice were 36.9 ± 6.6 and 2.0 ± 1.1, respectively. *P < 0.05. Error bars, mean ± SD. F. Detection of ADAM3 by immunoblotting. EMMPRIN was used as a control. Testis, whole testis; sperm, cauda epididymal spermatozoa. Het, heterozygous; DKO, *Prss51* and *Prss55* homozygous DKO. Black arrowheads, molecular weights (kDa). G. Imaging of sperm migration through the female reproductive tract. Wild-type females were mated with Het and DKO mice carrying the RBGS transgene, in which spermatozoa exhibit green fluorescence (EGFP) in the acrosome and red fluorescence (DsRed2) in the mitochondria within the flagellar midpiece. Upper panels, bright-field images; lower panels, red fluorescence images. Boxed regions (orange lines) of each left image indicate areas shown in higher magnification of each right image. White dashed lines, areas from the uterus to oviduct. Scale bars, 1 mm.

To determine the cause of infertility in DKO males, we performed IVF and found that the DKO spermatozoa from cauda epididymis were able to fertilize intact COCs from wild-type females (Figure 3A). We then performed IVF using oocytes without cumulus cells (cumulus-free) and oocytes without the ZP (ZP-free), both from wild-type females. A decline in the fertility rate was observed in DKO spermatozoa with cumulus-free (Figure 3B), but not with ZP-free oocytes (Figure 3C), suggesting that DKO spermatozoa have an abnormality in ZP interaction, rather than in sperm–oocyte fusion. We thus examined the ZP-binding ability of spermatozoa, using cumulus-free oocytes. As predicted, DKO spermatozoa barely bind to the ZP of wild-type oocytes (Figure 3D and E). Because these phenotypes in the DKO mice are similar to those in mice lacking sperm membrane protein ADAM3 (a disintegrin and metalloprotease 3) [27, 28], we performed immunoblot analysis to see whether ADAM3 protein levels are affected in the DKO spermatozoa.

ADAM3 protein is initially synthesized as a 110-kDa precursor in spermatids of the testis and processed into a 42-kDa mature protein in spermatozoa of the epididymis [29]. Whereas there was no difference in ADAM3 testicular protein levels between double Het and DKO males, the ADAM3 protein disappeared in DKO spermatozoa from cauda epididymis (Figure 3F).

Because ADAM3 is thought to play a pivotal role in sperm–ZP binding and sperm migration through the female reproductive tract [27, 28], we generated *Prss51* and *Prss55* DKO males carrying the RBGS transgene, mated them with wild-type females, and performed live imaging of the ejaculated spermatozoa inside the female reproductive tract. As predicted, the DKO spermatozoa barely migrate from the uterus to oviduct through the uterotubal junction (UTJ) (Figure 3G). These results indicate that *Prss51* and *Prss55* DKO spermatozoa have impaired ZP-binding ability in vitro and impaired sperm migration ability through the UTJ.

### Single KO mice lacking *Prss51* are fertile

**Figure 4:**
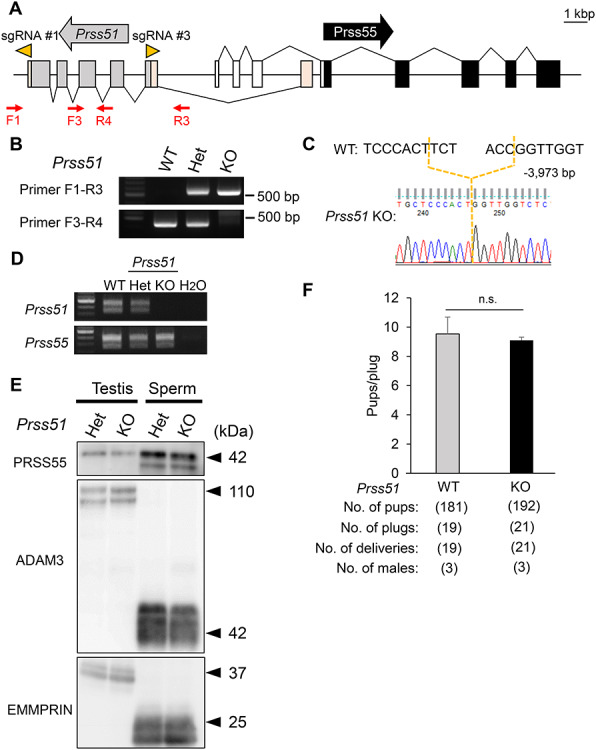
Male mice lacking *Prss51* are fertile. A. Genomic structure and KO strategy to create mouse *Prss51* KO mice. The direction of the arrows, in gray (*Prss51*) and black (*Prss55*), indicates the direction of transcription. The direction of the arrowheads (orange) indicates the direction of sgRNA (#1 and #3). Gray boxes, *Prss51* coding region; light orange boxes, *Prss51* noncoding region; black boxes, *Prss55* coding region; white boxes, *Prss55* noncoding region. F1 and F3, forward primers for genotyping; R3 and R4, reverse primers for genotyping. Scale bar, 1 kbp. B. Genotyping with genomic PCR. Primers shown in panel A were used for the PCR. WT, wild type; Het, heterozygous; KO, *Prss51* homozygous KO. C. DNA sequencing. The sequence of the PCR amplicon was analyzed. A region of 3973 bp was deleted in *Prss51* KO mice. D. Expression of mouse Prss51 and Prss55 was examined by RT-PCR using RNA isolated from testes. WT, wild type; Het, heterozygous; KO, *Prss51* homozygous KO. E. Detection of PRSS55 and ADAM3 by immunoblotting. EMMPRIN was used as a control. Testis, whole testis; sperm, cauda epididymal spermatozoa; Het, heterozygous; KO, *Prss51* homozygous KO. Black arrowheads, molecular weights (kDa). F. Fertility is presented as pups/plug: the number of total pups born divided by the number of vaginal plugs in females after natural mating. (9.5 ± 1.2 for WT females mated with WT males and 9.1 ± 0.2 with *Prss51* KO males). N.s., no significance (*P* > 0.05). Error bars, mean ± SD.

To determine whether *Prss51* is required for male fertility, we generated single KO mice lacking *Prss51*. Specifically, we injected both gRNAs and CAS9 enzyme into fertilized eggs to delete five exons of the *Prss51* gene that do not overlap with the *Prss55* gene locus, thereby preventing accidental disruption of *Prss55* gene (Figure 4A). We genotyped our mutant mice by genomic PCR (Figure 4B) and direct sequencing (Figure 4C). We examined the expression patterns of *Prss51* and *Prss55* by RT-PCR of testes from *Prss51* KO mice and found that *Prss51* expression was lost in the *Prss51* KO mouse testes, but that *Prss55* continued to be expressed (Figure 4D). We confirmed by immunoblot analysis that PRSS55 is expressed in *Prss51* KO testes and spermatozoa (Figure 4E), indicating that we successfully deleted the *Prss51* gene without impacting the *Prss55* gene.

We evaluated the fertility in wild-type and *Prss51* KO males (Figure 4F). The pregnancy rate and the average number of pups divided by the number of vaginal plugs did not differ significantly between wild-type and *Prss51* KO males, indicating that *Prss51* males are fertile. There was no difference in testicular morphology, weight, and histology (Supplementary Figure S4A–C), and in sperm morphology and motility (Supplementary Figure S4D and E), between *Prss51* Het and KO males. Moreover, *Prss51* KO spermatozoa were able to fertilize cumulus-intact oocytes from wild-type females by IVF (Supplementary Figure S4F), and there was no difference in testicular or sperm ADAM3 protein levels, between *Prss51* Het and KO males (Figure 4E). We conclude that *Prss51* is not required for male fertility in mice.

### Single KO Mice lacking *Prss55* gene have severe fertility defects

**Figure 5:**
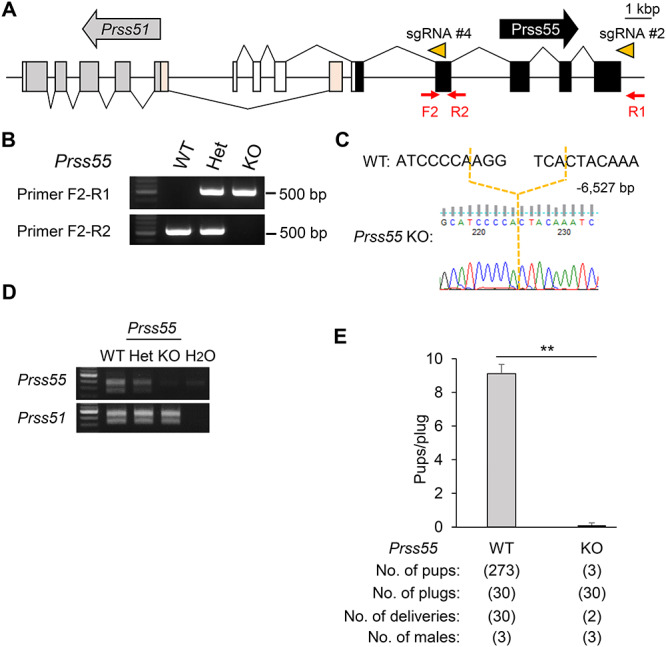
Male mice lacking *Prss55* have severe fertility defects. A. Genomic structure and KO strategy of mouse *Prss55* KO mice. The direction of the arrows, in gray (*Prss51*) and black (*Prss55*), indicates the direction of transcription. The direction of the arrowheads (orange) indicates the direction of sgRNA (#2 and #4). Gray boxes, *Prss51* coding region; light orange boxes, *Prss51* noncoding region; black boxes, *Prss55* coding region; white boxes, *Prss55* noncoding region. F2, forward primer for genotyping; R1 and R2, reverse primers for genotyping. Scale bar, 1 kbp. B. Genotyping with genomic PCR. Primers shown in panel A were used for PCR. WT, wild type; Het, heterozygous; KO, *Prss55* homozygous KO. C. DNA sequencing. The sequence of the PCR amplicon was analyzed. A region of 6527 bp was deleted in *Prss55* KO mice. D. The expression of mouse Prss51 and Prss55 was examined by RT-PCR using RNA isolated from testes. WT, wild type; Het, heterozygous; KO, *Prss55* homozygous KO. E. Fertility is presented pups/plug: the number of total pups born divided by the number of vaginal plugs in females after natural mating (9.1 ± 0.6 for WT females mated with WT males, and 0.1 ± 0.1 with *Prss55* KO males). **P < 0.01. Error bars, mean ± SD.

Because *Prss51* and *Prss55* DKO mice are sterile, but *Prss51* KO mice were fertile, we predicted that *Prss55* is essential or *Prss51 and Prss55* play redundant roles. To test this, we generated single KO mice lacking *Prss55*. We deleted four exons of the *Prss55* gene that do not overlap with *Prss51* (Figure 5A). The genotype of *Prss51* KO mice was confirmed by genomic PCR (Figure 5B) and direct sequencing (Figure 5C). We confirmed by RT-PCR that *Prss51*, but not *Prss55*, is expressed in *Prss55* KO testes (Figure 5D), indicating that we successfully deleted the *Prss55* gene without impacting *Prss51* gene expression.

**Figure 6:**
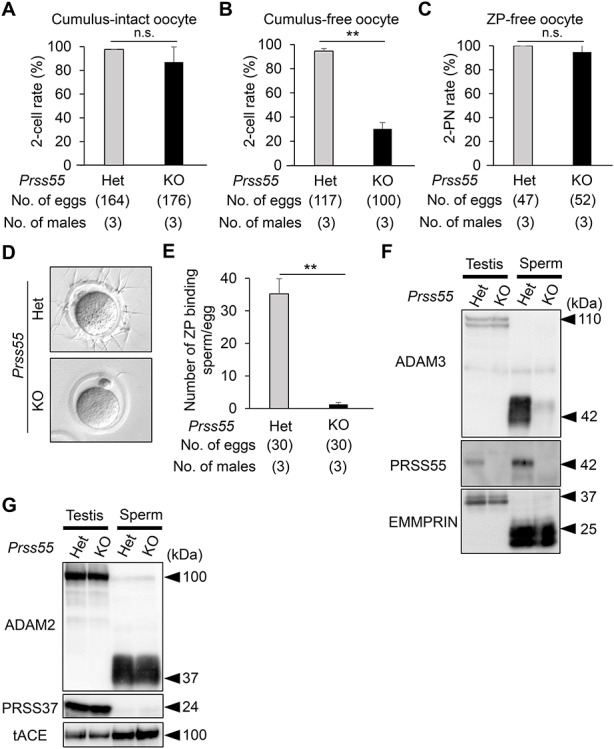
Phenotypic analysis of mouse spermatozoa lacking *Prss55*. A. IVF rates (percentage of two-cell stage eggs) in cumulus-intact oocytes using *Prss55* heterozygous (Het) and homozygous (KO) spermatozoa. Average IVF rates of Het and KO spermatozoa were 97.6 ± 0.1 and 86.9 ± 12.9%, respectively. N.s., no significance (*P* > 0.05). Error bars, mean ± SD. B. IVF rates (percentage of two-cell stage eggs) in cumulus-free oocytes using spermatozoa from Het and KO mice. Average IVF rates of Het and KO spermatozoa were 94.6 ± 2.2 and 30.2 ± 5.2%, respectively. **P < 0.01. Error bars, mean ± SD. C. IVF rates (percentage of two pronuclei [2PN] eggs) in ZP-free oocytes using spermatozoa from Het and KO mice. Average IVF rates of Het and KO spermatozoa were 100 and 94.7 ± 7.4%, respectively. N.s., no significance (*P* > 0.05). Error bars, mean ± SD. D. Sperm–ZP-binding assay. Spermatozoa from Het and KO mice were used to inseminate cumulus-free oocytes. Scale bars, 50 μm. E. Average numbers of ZP-binding spermatozoa per total number of eggs in Het and KO male mice were 35.2 ± 4.6 and 1.2 ± 0.6, respectively. **P < 0.01. Error bars, mean ± SD. F. Detection of ADAM3 and PRSS55 by immunoblotting. EMMPRIN was used as a control. Testis, whole testis; sperm, cauda epididymal spermatozoa; Het, heterozygous; KO, *Prss55* homozygous KO. Black arrowheads, molecular weights (kDa). G. Detection of ADAM2, PRSS37, and tACE by immunoblotting. EMMPRIN shown in panel E was used as a control. Testis, whole testis; sperm, cauda epididymal spermatozoa; Het, heterozygous; KO, *Prss55* homozygous KO. Black arrowheads, molecular weights (kDa).

We evaluated the fertility in wild-type and *Prss55* KO male mice (Figure 5E). The pregnancy rate (the number of deliveries per the number of the vaginal plugs) was 100% (30/30) for wild-type females mated with wild-type males, and 6.7% (2/30) for wild-type females mated with *Prss55* KO males (Figure 5E). The average number of pups divided by the number of vaginal plugs was 9.1 ± 0.6 for wild-type males (*n* = 3), and 0.1 ± 0.1 for *Prss55* KO males (*n* = 3), indicating that the *Prss55* KO has severe fertility defects (Figure 5E). There was no difference in testicular morphology, weight, and histology (Supplementary Figure S5A–C), and in sperm morphology and motility (Supplementary Figure S5D and E), between *Prss55* Het and KO males. Moreover, a decline in IVF rates was observed in *Prss55* KO spermatozoa with cumulus-free, but not with cumulus-intact or ZP-free oocytes (Figure 6A–C). We confirmed that the *Prss55* KO spermatozoa barely bind to the ZP of wild-type oocytes (Figure 6D and E) and that the ADAM3 protein signal disappeared in the *Prss55* KO spermatozoa (Figure 6F). Because ADAM3-associated proteins have been reported to affect ADAM3 protein and/or its processing or localization [29–31], we performed immunoblot analysis for these ADAM3-associated proteins, ADAM2, PRSS37, and tACE. We found that there was no difference in these protein levels between the Het and *Prss55* KO testis/spermatozoa (Figure 6G). Thus, we conclude that *Prss55* is required for male fertility in mice to affect ADAM3, independently of ADAM2, PRSS37, and tACE.

## Discussion

We performed functional analysis of two testis-enriched serine protease genes, *Prss51* and *Prss55*, which have partially overlapping gene loci in both mice and humans. By generating DKO and each single KO mice, we demonstrated that PRSS55, but not PRSS51, is essential for male fertility and sperm ADAM3 maturation.

Recent studies have questioned whether *Prss55* is required for male fertility. Although Shang et al. [8] showed that single KO male mice lacking *Prss55* exhibited severe male infertility, Khan et al. [9] showed that the *Prss55* KO males are fertile. We generated single KO mice lacking *Prss55* and found that the *Prss55* KO mice exhibited severe male infertility and sperm–ZP binding in vitro. These results are consistent with and support Shang et al.’s findings [8]. The difference between Khan et al.’s [9] findings and ours might be due to a difference in the KO strategy between producing a frameshift mutation and a deletion of multiple key exons, respectively. The indel mutation in Khan et al’s [9] study might have generated an alternative and/or new transcript that encodes a functional PRSS55 variant. Moreover, we found that *Prss51* KO males are fertile and that DKO males phenocopy *Prss55* KO males. Although DKO males are sterile, *Prss55* KO males sire a few offspring in our fertility test. It might be because *Prss55* KO males underwent a longer trial of fertility test than DKO males. Taken together, we conclude that *Prss55*, but not *Prss51*, is required for male fertility in mice.

The impaired sperm migration and impaired ZP-binding phenotypes, observed in *Prss55* KO mice, are shared among other KO mice that exhibit defects in ADAM3 protein [29]. KOs of >10 genes (*Ace-t*, *Adam1a*, *Adam2*, *Calr3*, *Clgn*, *Cmtm2a/b*, *Pdilt*, *Pmis2*, *Prss37*, *Rnase10*, *Tex101,* and *Tpst2*) and 2 gene clusters (*Cst* and *Pate*) have been reported to affect ADAM3 protein and/or its processing/localization in spermatozoa [29–31]. Indeed, we found that the ADAM3 protein signal disappeared in *Prss55* KO spermatozoa from cauda epididymis, suggesting that PRSS55 is required for ADAM3 maturation and subsequent sperm-fertilizing ability.

*Prss55* is exclusively expressed in testes, and no mRNA expression is detectable in epididymis [8]. Specifically, *Prss55* is expressed during late spermatogenesis [8, 9]. We confirmed that *Prss55* was expressed in testes from postnatal day 28 (Figure 1B), when elongating or elongated spermatids first appear in the testis [26]. We also found that PRSS55 protein is expressed in both testis and spermatozoa from cauda epididymis (Figure 6F), again consistent with previously reported findings [8]. These results suggest that PRSS55 is expressed in germ cells of the testis and retained in spermatozoa of the epididymis. In *Prss55* KO mice, although the ADAM3 signal disappeared in spermatozoa from cauda epididymis, ADAM3 protein levels were not affected in the testis. Thus, PRSS55 may have a pivotal function in epididymal spermatozoa, rather than testicular germ cells.

To generate *Prss51* and *Prss55* DKO mice, we deleted both genes in ES cells and found that the ES cells contributed to the germ cell lineage in chimeric mice (Supplementary Figure S2). The chimeric males contained spermatozoa derived from ES cells carrying a homozygous double deletion of *Prss51* and *Prss55*, which were transmitted to the offspring. Because *Prss51* and *Prss55* DKO mice are infertile, wild-type germ cells present in chimeric mice likely compensated for the function of *Prss55* in DKO spermatozoa. Thus, in the unperturbed wild-type testes, PRSS55 should be secreted/presented by germ cells and process ADAM3, extracellularly. It remains unclear how PRSS55 disrupts ADAM3 in spermatozoa. PRSS55 has no direct interaction with ADAM3 by immunoprecipitation with an anti-PRS55 antibody [8], indicating that PRSS55 functions to impact ADAM3 indirectly. We found that at least three proteins (ADAM2, PRSS37, and tACE), which have been reported to impact levels of ADAM3 [29, 30], showed no detectable changes in *Prss55* KO mice. Thus, PRSS55 might interact with other ADAM3-associated proteins [29–31], or proteins yet to be identified.

Elucidating the function of the testis-enriched genes is necessary to understand additional causes of male fertility and to develop male contraceptives. Because PRSS37 is exclusively expressed in testicular germ cells during late spermatogenesis and required for fertility in mice [15], PRSS37 is regarded as a potential target for the development of male contraceptive drugs. We propose that PRSS55 is a new potential target for contraceptive drugs for the following reasons. First, *Prss55* is specifically expressed in the testis of mice and humans. Testis-specific expression is suitable for a drug to inhibit the target proteins without any adverse effects. Second, PRSS55 functions extracellularly, suggesting that drugs that inhibit PRSS55 would not need to enter the cell to exert an inhibitory effect. Third, because PRSS55 is a putative trypsin-like serine protease, drugs can be developed to inhibit its putative enzymatic activity and/or block activation of its substrates and/or prevent loss of its substrates (i.e., substrates remain at a high level). Further functional analysis of PRSS55, such as the protease activity and the molecular mechanism of ADAM3 protein processing, will facilitate development of male or female contraceptive drugs.

## Author Contributions

KK, MMM, and MI designed the research; KK, TM, YL, and ZY performed the research; KK, TE, TM, MMM, and MI analyzed the data; and KK, TE, MMM, and MI wrote the paper.

## Supplementary Material

FigS1_20200214_ioaa041

FigS2_20200214_ioaa041

FigS3_20200214_ioaa041

FigS4_20200214_ioaa041

FigS5_20200214_ioaa041

Prss51_55_supplementary_data_20200214_final_ioaa041
