# Supplementary material for: Prss55 but not Prss51 is required for male fertility in mice
Source: Biol Reprod. 2020 Apr 17;103(2):223–34. doi: 10.1093/biolre/ioaa041 (PMC7401375; doi:10.1093/biolre/ioaa041)
Supplement: FigS1_20200214_ioaa041 [file figs1_20200214_ioaa041.pdf]

Fig. S1

A

```
h PRSS51   1 -MFQLLIPLLLALKGHAQDNPENVQCGHRPAFPNSSWLPFHERLQVQNGECPWQVSIQMS 59
m PRSS51   1 MMLPLLIALLMASKGQAKDQQESVLCGHRPAFPNSSWLPLRELLEVQHGEFPWQVSIQML 60

h PRSS51  60 RKHLCGGSILHWWWVLTAAHCFRRTLLDMAVVNVTVVMGTRTFSNIHSERKQVQKVIIHK 119
m PRSS51  61 GKHLCGGSIIHRWWVLTAAHCFPRTLLELVAVNVTVVMGIKTFSDTNLERKQVQKIIAHR 120

h PRSS51 120 DYKPPQLDSDLSLLLLATPVQFSNFKMPVCLQEEERTWDWCWMAQWVTTNGYDQYDDLNM 179
m PRSS51 121 DYKPPDLDSDLCLLLLATPIQFNKDKMPICLPQRENSWDRCWMSEWAYTHGHGSAKGSNM 180

h PRSS51 180 HLEKLRVVQISRKECAKRINQLSRNMICAWNEPGTNGIFKGDSGAPLVCAIYGTQRLFQV 239
m PRSS51 181 HLKKLRVVQISWRTCAKRVTQLSRNMLCAWKEVGTNGKCQGDSGAPMVCANWETRRLFQV 240

h PRSS51 240 GVFSGGIRSGSRGRPGMFVSVAQFIP---------------------------------- 265
m PRSS51 241 GVFSWGITSGSRGRPGIFVSVAQFIPWILEETQREGRALALSKASKSLLAGSPRYHPILL 300

h PRSS51 265 -------------------                                          265
m PRSS51 301 SMGSQILLAAIFSDDKSNC                                          319
```

B

```
h PRSS55   1 MLLFSVLLLLSLVTGTQLGPRTPLPEAGVAILGRARGAHRPQPPHPPSPVSECGDRSIFE 60
m PRSS55   1 MILPSILLLV---------AHT-L-EANV----------------------ECGVRPLYD 27

h PRSS55  61 GRTRYSRITGGMEAEVGEFPWQVSIQARSEPFCGGSILNKWWILTAAHCLYSEELFPEEL 120
m PRSS55  28 SRIQYSRIIEGQEAELGEFPWQVSIQESDHHFCGGSILSEWWILTVAHCFYAQELSPTDL 87

h PRSS55 121 SVVLGTNDLTSPSMEIKEVASIILHKDFKRANMDNDIALLLLASPIKLDDLKVPICLPTQ 180
m PRSS55  88 RVRVGTNDLTTSPVEL-EVTTIIRHKGFKRLNMDNDIALLLLAKPLTFNELTVPICLPLW 146

h PRSS55 181 PGPATWRECWVAGWGQTNAADKNSVKTDLMKAPMVIMDWEECSKMFPKLTKNMLCAGYKN 240
m PRSS55 147 PAPPSWHECWVAGWGVTNSTDKESMSTDLMKVPMRIIEWEECLQMFPSLTTNMLCASYGN 206

h PRSS55 241 ESYDACKGDSGGPLVCTPEPGEKWYQVGIISWGKSCGEKNTPGIYTSLVNYNLWIEKVTQ 300
m PRSS55 207 ESYDACQGDSGGPLVCTTDPGSRWYQVGIISWGKSCGKKGFPGIYTVLAKYTLWIEKIAQ 266

h PRSS55 301 LEGRPFNAEKRRTSVKQKPM-GSPVSGVPEPGSPRSWLLLCPLSHVLFRAILY--      352
m PRSS55 267 TEGKPLDFRGQSSSNKKKNRQNNQLSKSPALNCPQSWLLPCLLSFALLRALSNWK      321
```
