# Supplementary figures and images for: Prss55 but not Prss51 is required for male fertility in mice
Source: Biol Reprod. 2020 Apr 17;103(2):223–34. doi: 10.1093/biolre/ioaa041 (PMC7401375; doi:10.1093/biolre/ioaa041)

Fig. S2

**A**

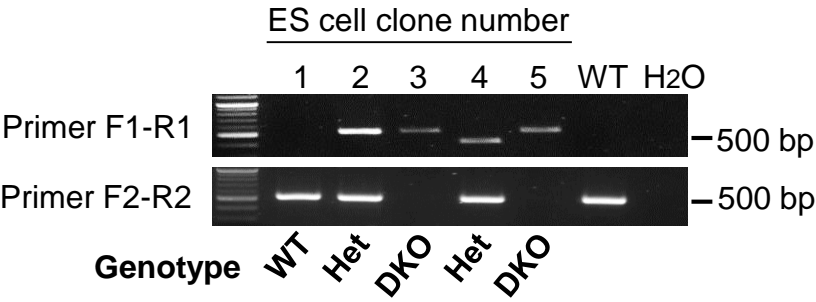

**B**

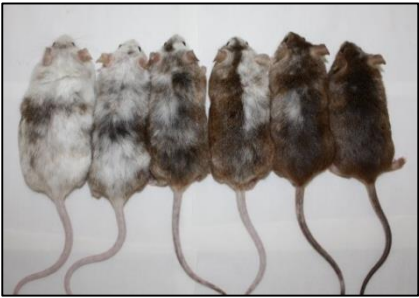

**C**

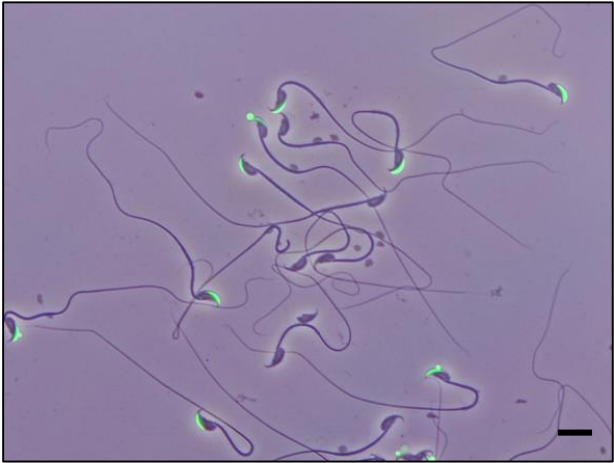

Supplement: FigS2_20200214_ioaa041 [file figs2_20200214_ioaa041.pdf]

Fig. S3

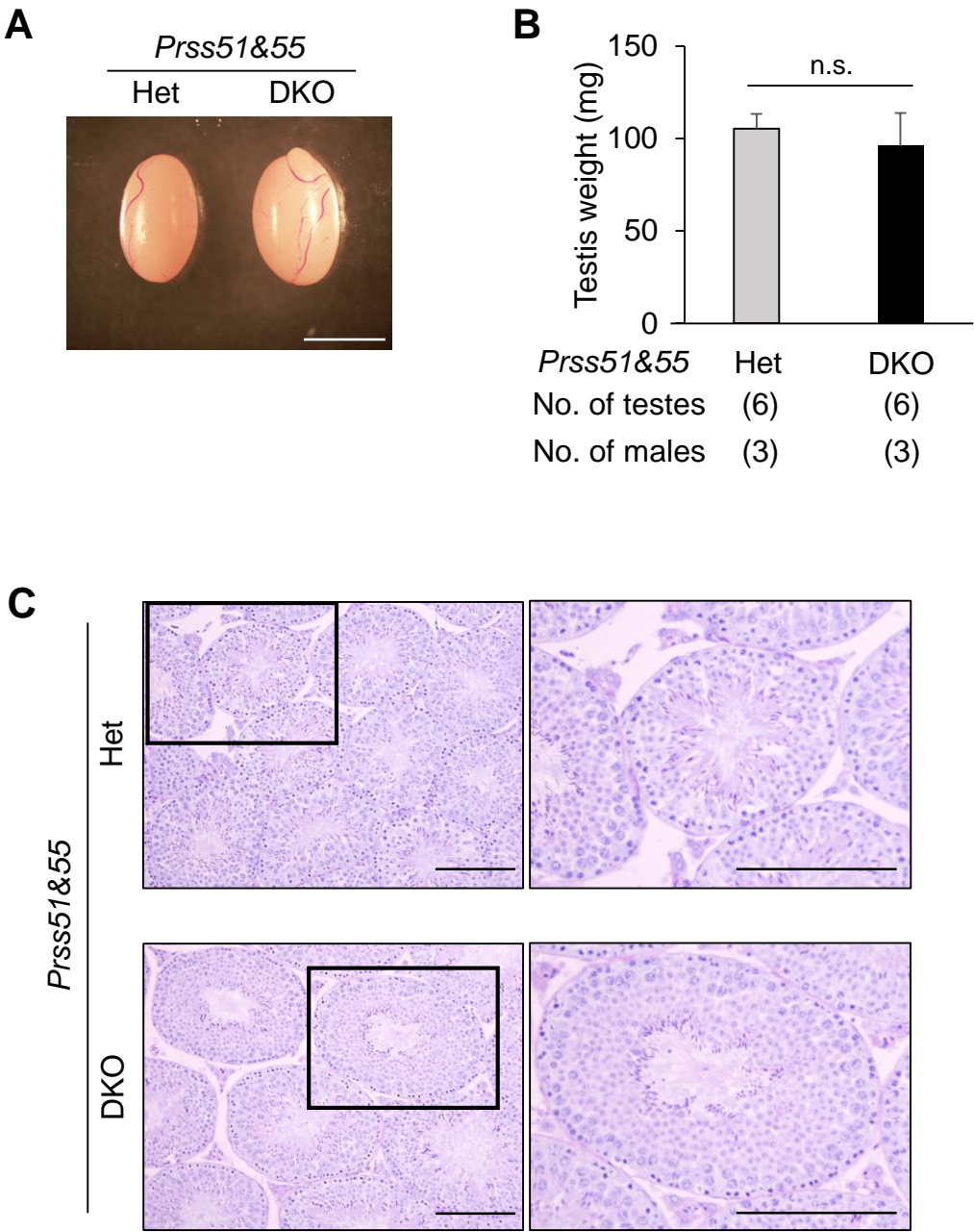

Supplement: FigS3_20200214_ioaa041 [file figs3_20200214_ioaa041.pdf]

**Fig. S4**

**A**

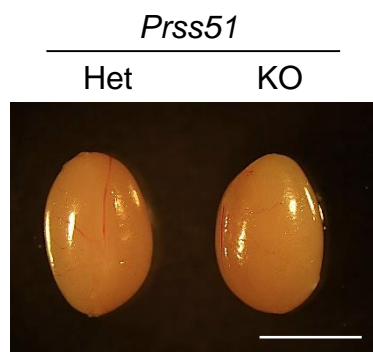

**B**

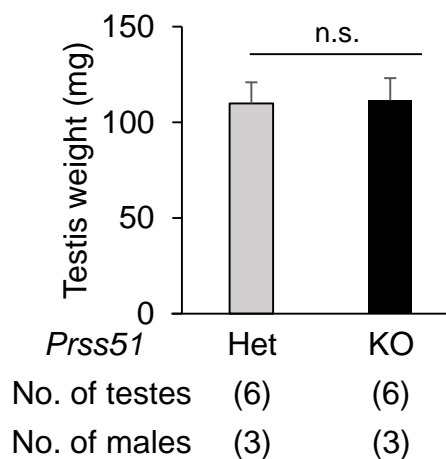

**C**

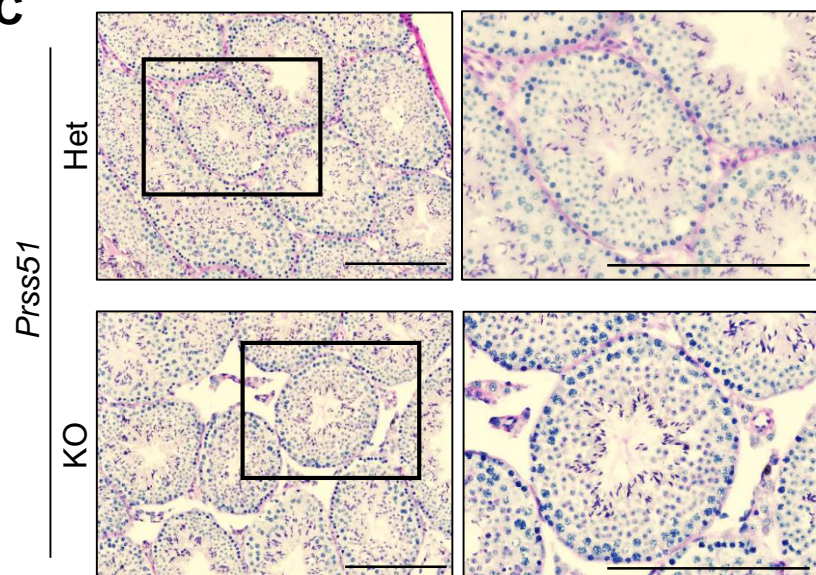

**D**

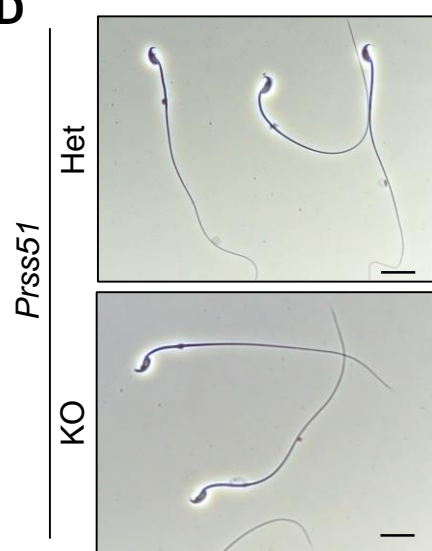

**E**

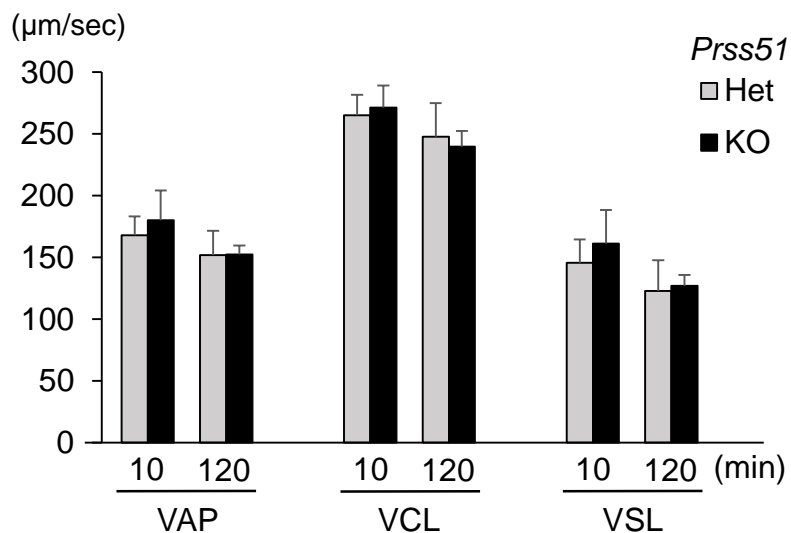

**F**

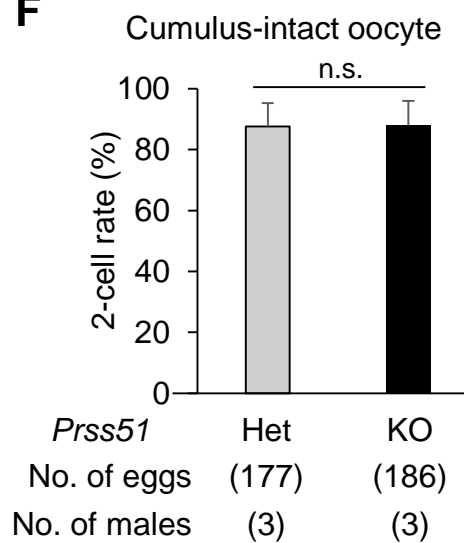

Supplement: FigS4_20200214_ioaa041 [file figs4_20200214_ioaa041.pdf]

**Fig. S5**

**A**

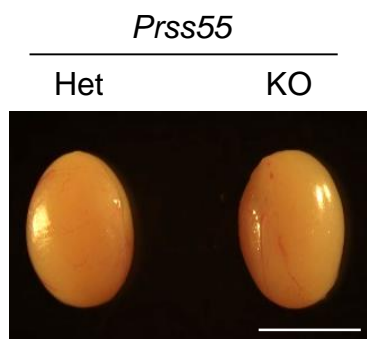

**B**

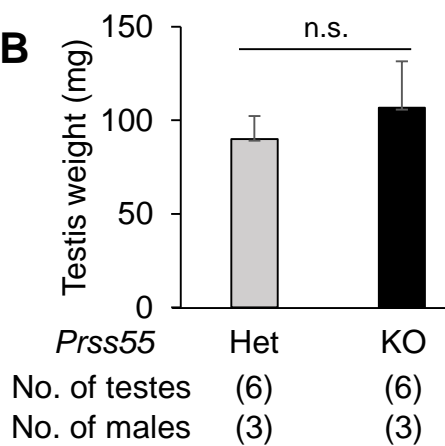

**C**

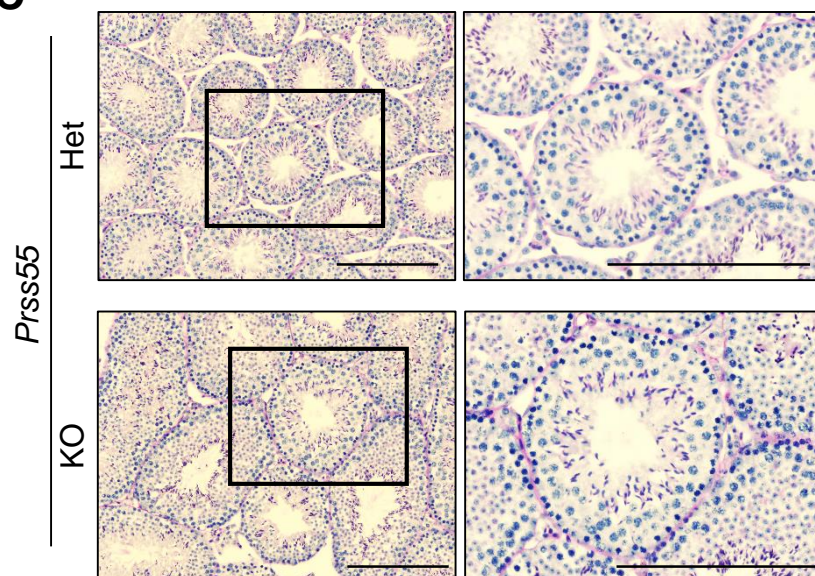

**D**

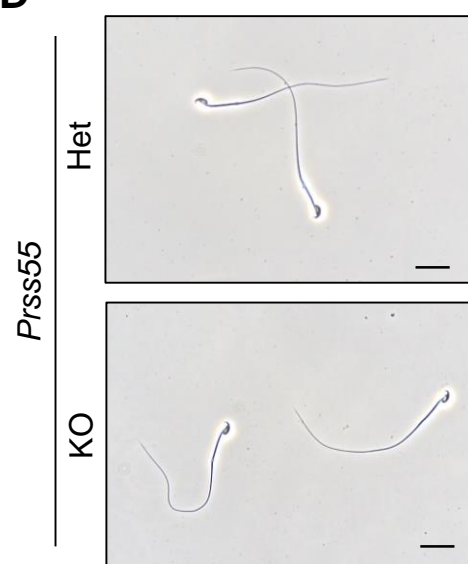

**E**

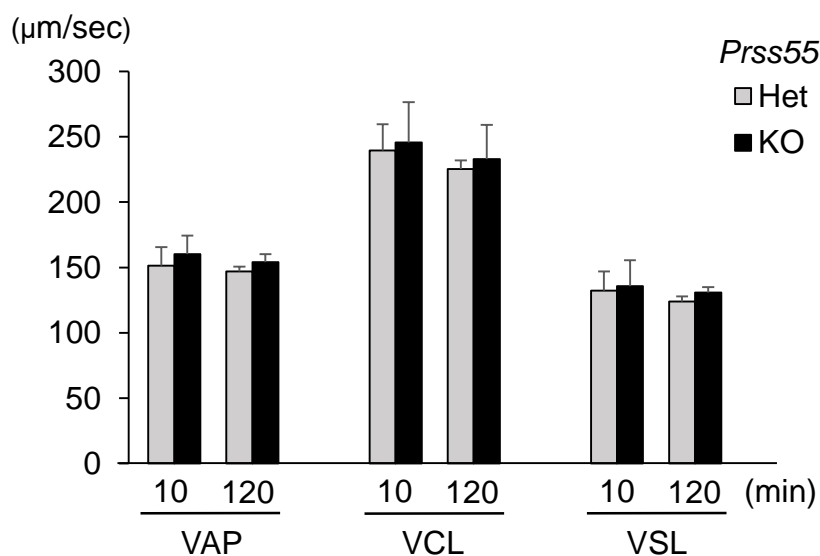

Supplement: FigS5_20200214_ioaa041 [file figs5_20200214_ioaa041.pdf]
