# Supplementary material for: Prss55 but not Prss51 is required for male fertility in mice
Source: Biol Reprod. 2020 Apr 17;103(2):223–34. doi: 10.1093/biolre/ioaa041 (PMC7401375; doi:10.1093/biolre/ioaa041)
Supplement: Prss51_55_supplementary_data_20200214_final_ioaa041 [file prss51_55_supplementary_data_20200214_final_ioaa041.docx]

**Supplementary Table S1.** Primers used for RT-PCR.

| **Gene** | **Forward primer (5’ to 3’)** | **Reverse primer (5’ to 3’)** | **Annealing** | **Elongation** | **Band size** |
| --- | --- | --- | --- | --- | --- |
| *Prss51* | AAGTCGACGCCGCCATG  ATGCTCCCACTTCTAAT | TTGAATTGGATTGGC  GTGGC | 65°C, 30 sec | 72°C, 30 sec | 362 bp or 457 bp |
| *Prss55* | AAGTCGACGCCGCCATG  ATCCTGCCCTCCATCCT | AAGTCGACTCCTGAG  CATAGAAGCAGTGG | 65°C, 30 sec | 72°C, 30 sec | 242 bp |
| *Actb* | CATCCGTAAAGACCTCT  ATGCCAAC | ATGGAGCCACCGATC  CACA | 65°C, 30 sec | 72°C, 30 sec | 171 bp |

**Supplementary Table S2.** Primers used for genotyping of mutant mice.

| **Gene** | **Allele** | **Primer name** | **Primer (5’ to 3’)** | **Annealing** | **Elongation** | **Band size** |
| --- | --- | --- | --- | --- | --- | --- |
| *Prss51*  and *55* | KO | F1 | AAGTCGACGAATGAACGGTCTC  ACGGTT | 65°C, 30 sec | 72°C, 30 sec | 466 bp |
|  |  | R1 | AAGTCGACGACTCCTTCATAGAG  AGGGA |  |  |  |
|  | WT | F2 | AAGCTAGCAGCTATTCGGTGGTC  AGCAGA | 65°C, 30 sec | 72°C, 30 sec | 525 bp |
|  |  | R2 | AAGTCGACTCCTGAGCATAGAA  GCAGTGG |  |  |  |
| *Prss51* | KO | F1 | AAGTCGACGAATGAACGGTCTC  ACGGTT | 65°C, 30 sec | 72°C, 30 sec | 434 bp |
|  |  | R3 | AAGCTAGCAGGCAGATGATGGT  TAGCATG |  |  |  |
|  | WT | F3 | CTTCACTCCAAAGCCACCAGG | 65°C, 30 sec | 72°C, 30 sec | 430 bp |
|  |  | R4 | GCTCTCAGTTACCTCAGGTGCC |  |  |  |
| *Prss55* | KO | F2 | AAGCTAGCAGCTATTCGGTGGTC  AGCAGA | 65°C, 30 sec | 72°C, 30 sec | 434 bp |
|  |  | R1 | AAGTCGACGACTCCTTCATAGAG  AGGGA |  |  |  |
|  | WT | F2 | AAGCTAGCAGCTATTCGGTGGTC  AGCAGA | 65°C, 30 sec | 72°C, 30 sec | 525 bp |
|  |  | R2 | AAGTCGACTCCTGAGCATAGAA  GCAGTGG |  |  |  |

**Supplementary Table S3.** List of antibodies used in this study.

| **Antigen** | **Type** | **Supplier (catalog no.) or source** | **Dilution** |
| --- | --- | --- | --- |
| ACE | Mouse-monoclonal | M. Ikawa (Osaka University) | 1:1000 |
| ADAM2 | Mouse-monoclonal | Merck (MAB19292) | 1:1000 |
| ADAM3 | Mouse-monoclonal | SantaCruz (sc-365288) | 1:1000 |
| EMMPRIN | Goat-polyclonal | SantaCruz (sc-9757) | 1:1000 |
| PRSS37 | Rabbit-  polyclonal | SIGMA (HPA020541) | 1:1000 |
| PRSS55 | Rabbit-  polyclonal | SIGMA (Custom) | 1:250 |
